# Interconnections between urolithiasis and oral health: a cross-sectional and bidirectional Mendelian randomization study

**DOI:** 10.3389/fmed.2023.1174502

**Published:** 2023-04-26

**Authors:** Jin-Zhou Xu, Jian-Xuan Sun, Lin-Tao Miao, Si-Han Zhang, Wen-Jie Wang, Chen-Qian Liu, Qi-Dong Xia, Jun-Lin Lu, Peng Zhou, Yong-Man Lv, Yang Xun, Wei Guan, Lei Cui

**Affiliations:** ^1^Department of Urology, Tongji Hospital, Tongji Medical College, Huazhong University of Science and Technology, Wuhan, China; ^2^Health Management Center, Tongji Hospital, Tongji Medical College, Huazhong University of Science and Technology, Wuhan, China

**Keywords:** oral–genitourinary axis, oral, urolithiasis, China, Mendelian randomization

## Abstract

**Introduction:**

Urolithiasis is one of the most common diseases for urologists and it is a heavy burden for stone formers and society. The theory of the oral–genitourinary axis casts novel light on the pathological process of genitourinary system diseases. Hence, we performed this study to characterize the crosstalk between oral health conditions and urolithiasis to provide evidence for prevention measures and mechanisms of stone formation.

**Materials and methods:**

This population-based cross-sectional study included 86,548 Chinese individuals who had undergone a comprehensive examination in 2017. Urolithiasis was diagnosed depending on the results of ultrasonographic imaging. Logistic models were utilized to characterize the association between oral health conditions and urolithiasis. We further applied bidirectional Mendelian randomization to explore the causality between oral health conditions and urolithiasis.

**Results:**

We observed that presenting caries indicated a negative correlation with the risk for urolithiasis while presenting gingivitis [OR (95% CI), 2.021 (1.866–2.187)] and impacted tooth [OR (95% CI), 1.312 (1.219–1.411)] shown to be positively associated with urolithiasis. Furthermore, we discovered that genetically predicted gingivitis was associated with a higher risk of urolithiasis [OR (95% CI), 1.174 (1.009–1.366)] and causality from urolithiasis to impacted teeth [OR(95% CI), 1.207 (1.027–1.418)] through bidirectional Mendelian randomization.

**Conclusion:**

The results cast new light on the risk factor and pathogenesis of kidney stone formation and could provide novel evidence for the oral–genitourinary axis and the systematic inflammatory network. Our findings could also offer suggestions for tailored clinical prevention strategies against stone diseases.

## Key messages

1. We provided novel evidence for the oral–genitourinary axis and the systematic inflammatory network.

2. We discovered that aged individuals with poor oral health were more vulnerable to urolithiasis.

3. Our results cast new light on the pathogenesis of kidney stone formation and offer suggestions for accurate clinical prevention and treatment strategies against stone diseases.

## Introduction

Urolithiasis is one of the most common diseases for urologists that affects between 10 and 15% of the world's population (1). Stone formers suffer from symptoms, high recurrence rate, financial burden, and increased risk of hydronephrosis, renal dysfunction, and renal cell carcinoma (2). Hence, there is an imperious need to elaborate on the factors affecting the stone formation and accurate prevention strategies against urolithiasis.

Recently, an oral–genitourinary axis has been proposed. Periodontitis was identified as a potential risk of genitourinary cancers, especially prostate cancer and bladder cancer (3). A cohort study examined the risk of cancer in patients with chronic periodontitis and found that chronic periodontitis was significantly and positively associated with the risk of prostate cancer (4). In non-smokers, advanced periodontitis was reported to be associated with an elevated risk of bladder cancer (5). Other publications also characterized the correlation of oral health conditions with benign prostatic hyperplasia, chronic kidney disease, and lower urinary tract symptoms (6). Furthermore, Estemalik et al. reported the presence of similar bacterial DNA in both the genitourinary system and oral cavity from the same individual (7). Accordingly, a recent study from Fang et al. suggested that the oral microbiome could be the potential target for the management of the genitourinary disease (8). These publications broadened the horizon to unravel the pathophysiology of diseases of the genitourinary system. However, there is a little study to explore the association between oral health conditions and urolithiasis, which are both closely related to inflammatory status and abnormal osteogenesis.

Mendelian randomization (MR) study is a method to assess the causal effect of an exposure on an outcome using an instrument, single-nucleotide polymorphisms (SNPs), as a proxy for the exposure (9). This technique can to a considerable extent eliminate residual confounding and has been applied to explore the causality in multiple diseases, including urolithiasis. Yuan et al. provided genetic evidence in support of causal associations of coffee and caffeine consumption with urolithiasis via MR design (10).

Hence, we performed this study to characterize the crosstalk and the causal association between oral health conditions and urolithiasis. The results were hopeful to cast new light on the risk factor and pathogenesis of kidney stone formation and could provide novel evidence for the oral–genitourinary axis. Our findings could also offer suggestions for accurate clinical prevention strategies against stone diseases.

## Methods

### Study population

This research was part of the series of research Influencing Factors for Common Chronic Diseases among the Chinese Population (IFCCDCP). The participants (*n* = 99,859) have undergone comprehensive examinations in the physical examination center of Tongji Hospital, Tongji Medical College, Huazhong University of Science and Technology, in 2017. This study was approved by the institutional review board of Tongji Hospital, Tongji Medical College, Huazhong University of Science and Technology (Approval ID: TJ-C20160115). The study conformed to the ethical guidelines of the Declaration of Helsinki. Consent was waived by the ethics committee.

**Figure 1:**
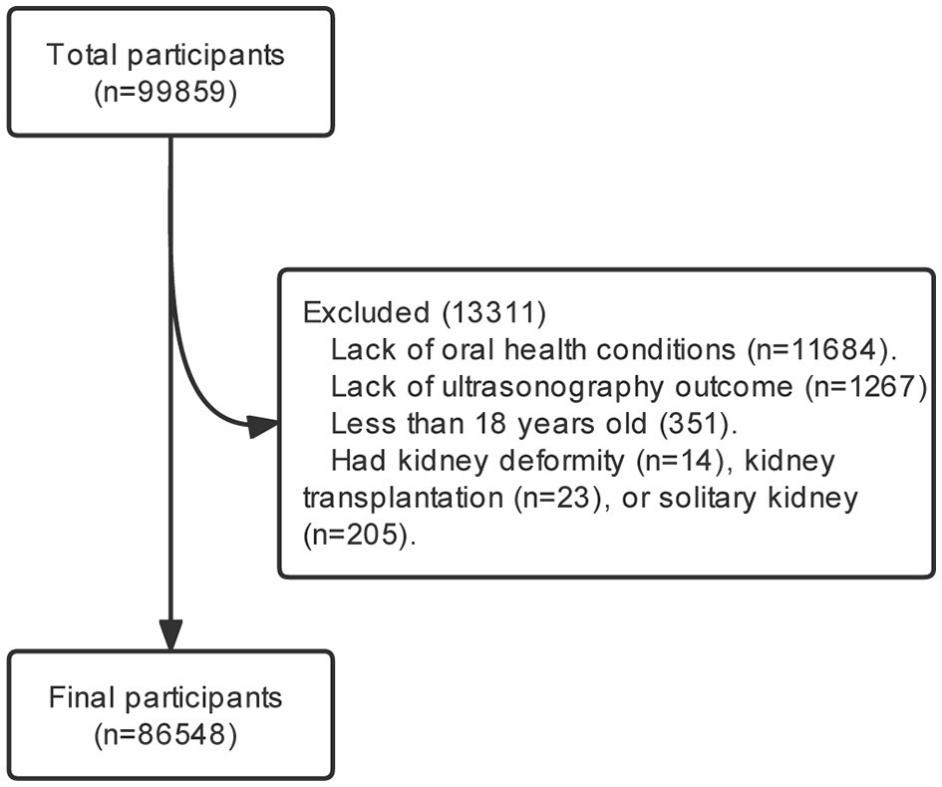
The flow of study participants in the study. After excluding 13,311 participants who were lack of oral health information (*n* = 11,684), ultrasonography outcome (*n* = 1,267), were <18 years old (*n* = 351), or had kidney deformity (*n* = 14), kidney transplantation (*n* = 23), solitary kidney (*n* = 205), those who completed a health examination were recruited (*n* = 86,548).

After excluding 13,311 participants who were lack of oral health information (*n* = 11,684), ultrasonography outcome (*n* = 1,267), were <18 years old (*n* = 351), or had kidney deformity (*n* = 14), kidney transplantation (*n* = 23), and solitary kidney (*n* = 205), a total of 86,548 participants were included (Figure 1).

The Mendelian randomization (MR) study was based on summary-level data from the FinnGen consortia (11). It is a public–private partnership combining genotype data from Finnish biobanks and digital health record data from Finnish health registries. In the dataset, we included the participant information on urolithiasis (with 5,347 cases and 213,445 non-cases), dental caries (with 4,170 cases and 195,395 non-cases), gingivitis and periodontal diseases (with 4,120 cases and 195,395 non-cases), and impacted teeth (with 884 cases and 217,908 non-cases) (Supplementary Table 1).

### Measurement

Urolithiasis was diagnosed depending on the results of ultrasonographic imaging. Oral health conditions, including caries, residual root or crown, denture, dental calculi, dental plaque, gingivitis, and impacted tooth, were determined by experienced dentists through full-mouth clinical examination. In FinnGen, cases were defined by N20 in the International Classification of Diseases, 10th Revision (ICD-10), and 592 in ICD-8 and ICD-9.

Demographic characteristics (sex and age) and comorbidities, including hypertension (HBP), diabetes mellitus (DM), coronary heart disease (CHD), and fatty liver (FL), were collected based on medical history. Height, weight [based on which body mass index (BMI) was calculated], and blood pressure [systolic blood pressure (SBP) and diastolic blood pressure (DBP)] were measured during the examination. Laboratory outcomes were analyzed from blood and urine specimens, including total protein (TP), albumin (Alb), globulin (Glo), serum creatinine (SCr), total cholesterol (TC), high-density lipoprotein cholesterol (HDL), low-density lipoprotein cholesterol (LDL), triglycerides (TG), uric acid (UA), fasting glucose (Glu), and urine pH (UpH). CKD-EPI China equation with an adjusted coefficient of 1.1 for the Chinese population was applied to calculate eGFR, where κ is 0.7 for women and 0.9 for men, α is −0.329 for women and −0.411 for men, min is the minimum of SCr/κ or 1, and max indicates the maximum of SCr/κ or 1 (12):


$$\begin{array}{l} eGFR=141\;*\;\min{\left(\frac{SCr}{\kappa },1\right)}^{\alpha }*\;\max{\left(\frac{SCr}{\kappa },1\right)}^{-1.209}*\\ \;0.993^{Age}*1.018\left(if\;female\right)*1.1 \end{array}$$

### Statistical analyses

Multiple imputation method was applied to process the missing values to increase statistical test efficiency and reduce bias. Categorical and continuous data were presented as number (percentage) and mean ± standard deviation. Chi-squared tests and Student's *t*-tests were applied for descriptive analyses.

Logistic regression models were used to evaluate the association between oral health conditions and the occurrence of urolithiasis. The outcomes were presented as odds ratio (OR) [95% confidence interval (CI)]. Models were adjusted for oral health conditions (Model 1), demographic characteristics and comorbidities (age, sex, BMI, HBP, DM, CHD, and FL) (Model 2), and laboratory outcomes (Alb, Glo, eGFR, HDL, LDL, TG, UA, Glu, and UpH) (Model 3). Model 3 was the primary model. In subgroup analyses, variables were grouped as follows: sex (male and female); age (18–29, 30–44, 45–59, and ≥60 y); BMI (≤ 18.5, 18.5–23.9, 24.0–27.9, and ≥28 kg/m^2^). Tests for interaction were performed using Wald's test.

In the MR study, single-nucleotide polymorphisms (SNPs) were determined as instrumental variables for urolithiasis, dental caries, gingivitis and periodontal diseases, and impacted teeth (*p* < 5 × 10^−6^, *r*^2^ <0.001 and clump distance >5,000 kb). The inverse-variance weighted (IVW) method was the primary statistical model in the MR study. Random-effects method was applied to evaluate the causal association. The pleiotropic effects were tested by the pleiotropy test, and a *p*-value > 0.05 was regarded to manifest no pleiotropic effects. MR-Egger was applied when pleiotropy was detected (*p* < 0.05). Multivariable MR was applied to further explore causal links. *Q*-value was used to assess the heterogeneity. The leave-one-out strategy was applied for sensitivity analysis.

R software (version 4.0.3) was used to perform all the statistical analyses. All *P*-values were two-tailed. A *P*-value <0.05 was considered statistically significant.

## Results

We compared the oral health conditions, presenting characteristics, comorbidities, and laboratory outcomes between those presented with and those without urolithiasis. Among the 86,548 adult participants (aged 18–96 years), 9,819 (11.41%) individuals were diagnosed to present urolithiasis according to ultrasonographic imaging outcomes. Approximately 56.2% of the study sample was male participants, and the overall mean age of the sample was 41.16 ± 12.83 (Table 1).

**Table 1:** Basic characteristics of the included participants with or without KS.

**Variables**	**All participants**	**Participants without KS^a^**	**Participants with KS^a^**	***p-*value**
	***n*** = **86,548**	***n*** = **76,729**	***n*** = **9,819**	
**Oral health conditions**				
Caries, present (%)	21,660 (25.0)	19,488 (25.4)	2,172 (22.1)	<0.001
Residual root or crown, present (%)	8,116 (9.4)	7,056 (9.2)	1,060 (10.8)	<0.001
Denture, present (%)	7,130 (8.2)	6,305 (8.2)	825 (8.4)	0.543
Dental calculus, present (%)	32,425 (37.5)	28,433 (37.1)	3,992 (40.7)	<0.001
Dental plaque, present (%)	8,271 (9.6)	7,111 (9.3)	1,160 (11.8)	<0.001
Gingivitis, present (%)	5,242 (6.1)	4,095 (5.3)	1,147 (11.7)	<0.001
Impacted tooth, present (%)	10,468 (12.1)	9,027 (11.8)	1,441 (14.7)	<0.001
**Presenting characteristics and comorbidities**				
Sex, male (%)	48,678 (56.2)	41,599 (54.2)	7,079 (72.1)	<0.001
Age, y	41.16 ± 12.83	40.80 ± 12.83	43.93 ± 12.52	<0.001
BMI, kg/m^2^	23.55 ± 3.39	23.46 ± 3.38	24.30 ± 3.31	<0.001
Hypertension, present (%)	7,284 (8.4)	6,003 (7.8)	1,281 (13.0)	<0.001
Diabetes, present (%)	2,097 (2.4)	1,751 (2.3)	346 (3.5)	<0.001
Coronary heart disease, present (%)	468 (0.5)	391 (0.5)	77 (0.8)	0.001
Fatty liver, present (%)	23,203 (26.8)	19,637 (25.6)	3,566 (36.3)	<0.001
SBP, mmHg	123.67 ± 17.86	123.22 ± 17.70	127.18 ± 18.65	<0.001
DBP, mmHg	75.68 ± 11.98	75.35 ± 11.85	78.29 ± 12.60	<0.001
**Laboratory parameters**				
TP, g/L	76.01 ± 3.94	76.02 ± 3.93	75.87 ± 4.00	<0.001
Alb, g/L	46.13 ± 2.59	46.14 ± 2.58	46.10 ± 2.62	0.137
Glo, g/L	29.87 ± 3.54	29.89 ± 3.54	29.77 ± 3.59	0.003
Scr, μmol/L	73.74 ± 18.65	73.11 ± 18.41	78.68 ± 19.75	<0.001
eGFR, mL/min/1.73 m^2b^	112.18 ± 17.17	112.76 ± 16.96	107.59 ± 18.02	<0.001
TC, mmol/L	4.52 ± 0.87	4.51 ± 0.86	4.61 ± 0.88	<0.001
HDL, mmol/L	1.28 ± 0.31	1.29 ± 0.31	1.23 ± 0.29	<0.001
LDL, mmol/L	2.72 ± 0.74	2.71 ± 0.74	2.81 ± 0.76	<0.001
TG, mmol/L	1.45 ± 1.26	1.43 ± 1.24	1.65 ± 1.41	<0.001
UA, mg/dL	341.53 ± 95.35	337.96 ± 93.81	369.40 ± 102.43	<0.001
Glu, mmol/L	5.31 ± 1.09	5.29 ± 1.07	5.45 ± 1.23	<0.001
UpH	6.12 ± 0.65	6.13 ± 0.65	6.10 ± 0.64	<0.001

KS, kidney stone; BMI, body mass index; SBP, systolic blood pressure; DBP, diastolic blood pressure; TP, total protein; Alb, albumin; Glo, globulin; SCr, serum creatinine; eGFR, estimated glomerular filtration rate; TC, total cholesterol; HDL, high-density lipoprotein cholesterol; LDL, low-density lipoprotein cholesterol; TG, triglycerides; UA, uric acid; Glu, fasting glucose; UpH, Urine pH.

^a^KS was diagnosed depending on the results of ultrasonographic imaging.

^b^Calculated using the CKD-EPI equation (details can be found in Methods section).

**Figure 2:**
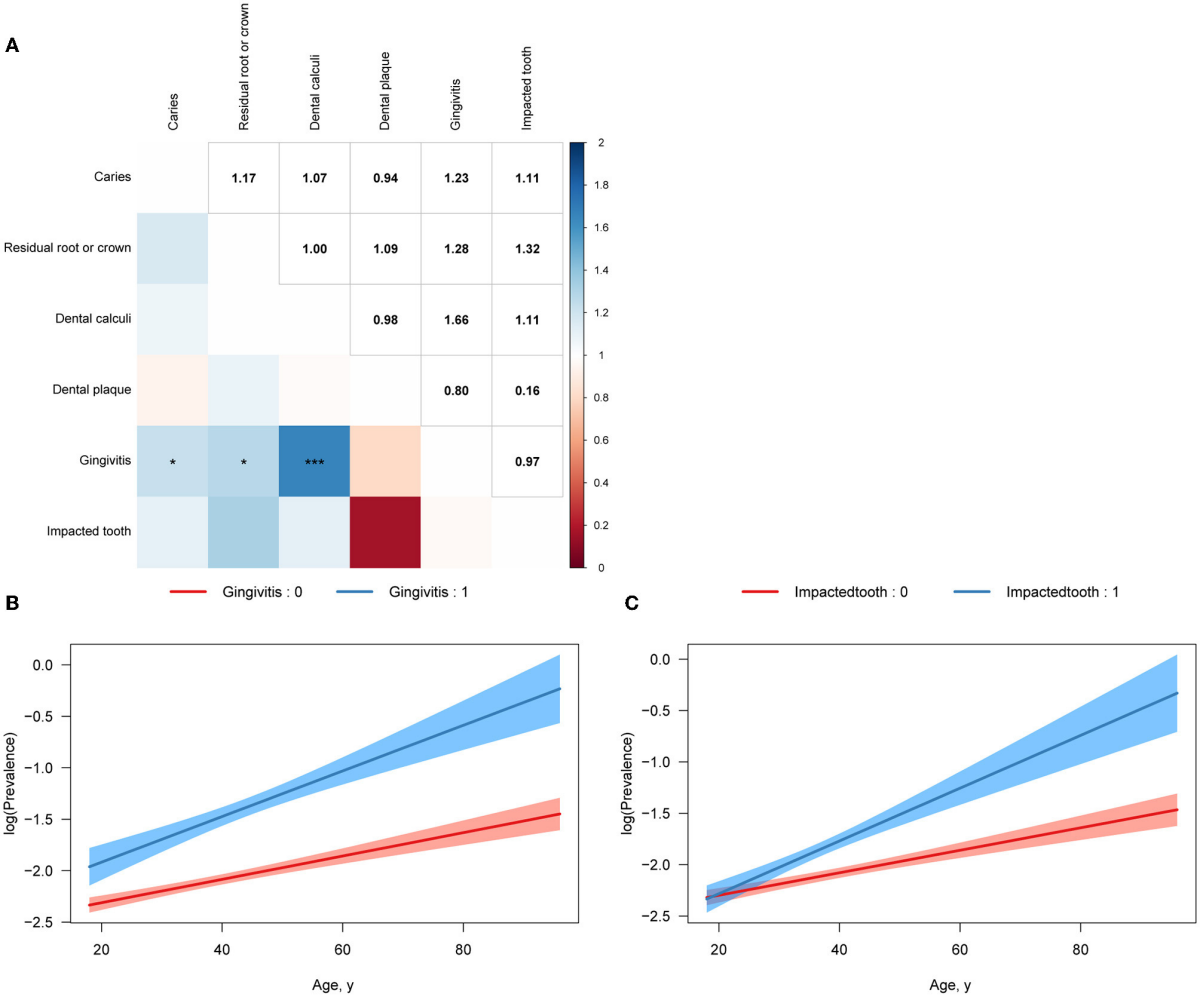
Illustrations of the interaction. Adjusted as Model 3 (see Methods—Statistical analyses section for descriptions of Model 3). **(A)** Illustration showed interaction among oral health conditions. The color and figures presented the odds ratio (OR) of the interaction term. *P* for interaction was calculated by applying Wald's test. ^*^p < 0.05; ^**^p < 0.01; ^***^p < 0.001. **(B)** Illustration showed the interaction between age and the gingivitis condition. **(C)** Illustration showed the interaction between age and the impacted tooth condition.

We then applied logistic regression to investigate the association between oral health conditions and the presence of urolithiasis. The ORs (95% CI) for urolithiasis of caries were 0.907 (0.861–0.955), 0.919 (0.872–0.969), and 0.908 (0.858–0.960) after adjusting for Model 1, Model 2, and Model 3, respectively. The ORs (95% CI) for urolithiasis of gingivitis were 2.247 (2.090–2.414), 1.937 (1.799–2.085), and 2.021 (1.866–2.187) after adjusting for Model 1, Model 2, and Model 3, respectively. The ORs (95% CI) for urolithiasis of impacted tooth were 1.305 (1.224–1.390), 1.311 (1.226–1.400), and 1.312 (1.219–1.411) after adjusting for Model 1, Model 2, and Model 3, respectively (Table 2). Additionally, we assess the interaction effect of oral health conditions. The ORs (95% CI) for the interaction effect of gingivitis with caries, residual root or crown, and dental calculi were 1.229 (1.010–1.489), 1.279 (1.029–1.584), and 1.663 (1.416–1.959), respectively (Figure 2A).

**Table 2:** Association between oral health conditions and urolithiasis using an extended model approach.

	**Univariate**	**Model 1-adjusted**	**Model 2-adjusted**	**Model 3-adjusted**
**Odds ratio (95% CI)**				
Caries	0.834 (0.793 ~ 0.877)^***^	0.907 (0.861 ~ 0.955)^***^	0.912 (0.866 ~ 0.961)^***^	0.912 (0.865 ~ 0.961)^***^
Residual root or crown	1.195 (1.116 ~ 1.279)^***^	1.211 (1.129 ~ 1.297)^***^	0.999 (0.930 ~ 1.073)	1.007 (0.937 ~ 1.081)
Dental calculus	1.164 (1.115 ~ 1.215)^***^	1.134 (1.083 ~ 1.187)^***^	0.985 (0.940 ~ 1.032)	0.991 (0.946 ~ 1.039)
Dental plaque	1.312 (1.227 ~ 1.400)^***^	1.492 (1.393 ~ 1.597)^***^	0.934 (0.869 ~ 1.004)	0.939 (0.873 ~ 1.010)
Gingivitis	2.346 (2.188 ~ 2.513)^***^	2.247 (2.090 ~ 2.414)^***^	1.925 (1.788 ~ 2.070)^***^	1.932 (1.794 ~ 2.078)^***^
Impacted tooth	1.290 (1.214 ~ 1.369)^***^	1.305 (1.224 ~ 1.390)^***^	1.312 (1.229 ~ 1.401)^***^	1.302 (1.219 ~ 1.390)^***^

Models:

Model 1: adjusted for oral health conditions (caries, residual root or crown, dental calculus, dental plaque, gingivitis, and impacted tooth).

Model 2: Model 1 plus demographic characteristics and comorbidities (age, sex, BMI, HBP, DM, CHD, and FL).

Model 3: Model 2 plus laboratory outcomes (Alb, Glo, eGFR, HDL, LDL, TG, UA, Glu, and UpH).

BMI, body mass index; HBP, hypertension; DM, diabetes mellitus; CHD, coronary heart disease; FL, fatty liver; Alb, albumin; Glo, globulin; SCr, serum creatinine; eGFR, estimated glomerular filtration rate; HDL, high-density lipoprotein cholesterol; LDL, low-density lipoprotein cholesterol; TG, triglycerides; UA, uric acid; Glu, fasting glucose; UpH, Urine pH; CI, confidence interval.

^*^p < 0.05; ^**^p < 0.01; ^***^p < 0.001.

We conducted subgroup analyses to further investigate the association between oral health conditions and urolithiasis. The ORs (95% CI) for gingivitis in those who were ≤ 29, 30–44, 45–59, and ≥60 years old were 1.827 (1.440–2.294), 1.648 (1.459–1.856), 2.106 (1.877–2.361), and 2.154 (1.679–2.746), respectively, which showed an increasing trend as age increases (*p* for interaction <0.001). The ORs (95% CI) for the impacted tooth in those who were 18–29, 30–44, 45–59, and ≥60 years old were 1.234 (1.074–1.415), 1.283 (1.169–1.407), 1.397 (1.215–1.602), and 1.481 (0.933–2.282), which also indicated an increasing trend as age increases (*p* for interaction <0.001) (Table 3). Furthermore, we investigated the interaction effect of age with gingivitis and impacted tooth in a visualization method. It was observed that the coexistence of aging and gingivitis or impacted tooth had a synergistic effect on urolithiasis (Figures 2B, C). The ORs (95% CI) for gingivitis in those whose BMI were ≤ 18.5, 18.5–23.9, 24.0–27.9, and ≥28 kg/m^2^ were 1.390 (0.803–2.270), 1.747 (1.554–1.960), 2.038 (1.819–2.281), and 2.265 (1.865–2.744), presenting a higher positive relation between gingivitis and urolithiasis as BMI increasing (*p* for interaction = 0.005) (Supplementary Table 2). We also separately explored the association between oral health conditions and urolithiasis in both genders and observed a closer link between an impacted tooth and urolithiasis in males [OR (95% CI) 1.159 (1.013–1.322) vs. OR (95% CI) 1.361 (1.261–1.468), *p* for interaction = 0.037] (Supplementary Table 3; Supplementary Figure 1). We applied logistic regression using the data with missing information in sensitivity analyses after adjusting for Model 1, Model 2, and Model 3, which confirmed the study results (Supplementary Table 4).

**Table 3:** Association between oral health conditions and urolithiasis grouped by age.

	**Age ≤ 29**	**29 <age ≤ 44**	**44 <age ≤ 59**	**59 <age**	***p*-for interaction**
**Odds ratio (95% CI)**					
Caries	0.901 (0.767 ~ 1.053)	0.909 (0.834 ~ 0.990)^*^	0.911 (0.835 ~ 0.992)^*^	0.908 (0.788 ~ 1.044)	0.050
Residual root or crown	0.742 (0.502 ~ 1.056)	0.994 (0.860 ~ 1.143)	1.102 (0.991 ~ 1.223)	1.005 (0.866 ~ 1.164)	0.039^*^
Dental calculus	0.845 (0.731 ~ 0.975)^*^	1.008 (0.935 ~ 1.087)	0.951 (0.879 ~ 1.028)	0.960 (0.837 ~ 1.101)	0.779
Dental plaque	0.915 (0.581 ~ 1.377)	0.867 (0.760 ~ 0.987)^*^	0.914 (0.824 ~ 1.014)	0.878 (0.704 ~ 1.089)	0.092
Gingivitis	1.827 (1.440 ~ 2.294)^***^	1.648 (1.459 ~ 1.856)^***^	2.106 (1.877 ~ 2.361)^***^	2.154 (1.679 ~ 2.746)^***^	<0.001^***^
Impacted tooth	1.234 (1.074 ~ 1.415)^**^	1.283 (1.169 ~ 1.407)^***^	1.397 (1.215 ~ 1.602)^***^	1.481 (0.933 ~ 2.282)	<0.001^***^

CI, confidence interval.

Model 3 was applied (see Methods—Statistical analyses section for descriptions of Model 3).

^*^p < 0.05; ^**^p < 0.01; ^***^p < 0.001.

**Figure 3:**
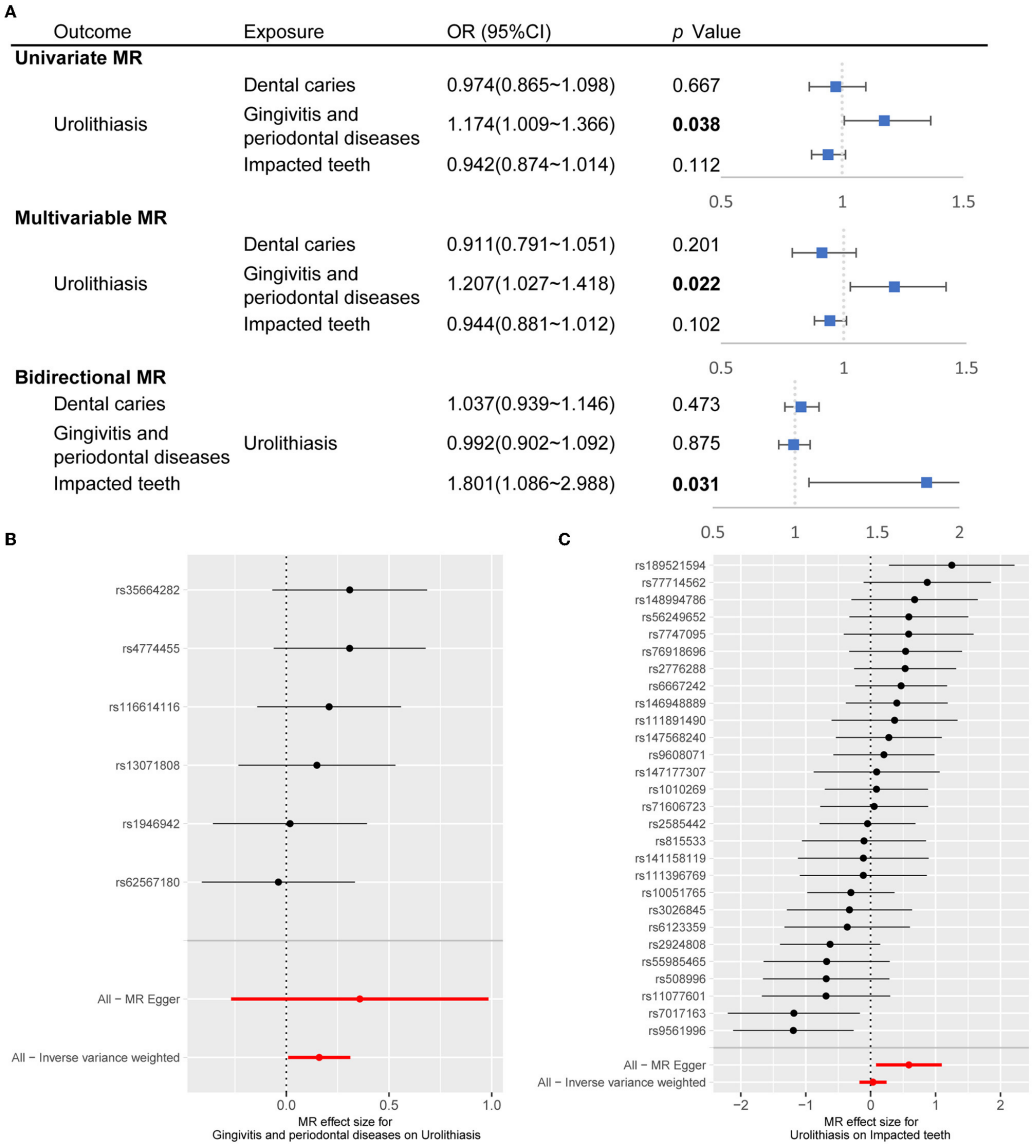
Causal association between oral conditions and urolithiasis through MR study. **(A)** Forest plot showed OR (95% CI) through univariate MR, multivariable MR, and bidirectional MR. The inverse-variance weighted was the major method. The OR for urolithiasis on impacted teeth applied MR-Egger since pleiotropy was detected. **(B)** Forest plot showed the effect of instrumental SNPs of gingivitis and periodontal diseases on urolithiasis. **(C)** Forest plot showed the effect of instrumental SNPs of urolithiasis on impacted teeth. MR, Mendelian randomization; SNP, single-nucleotide polymorphisms; OR, odds ratio; CI, confidence interval.

Mendelian randomization (MR) study is a method to assess the causal effect of an exposure on an outcome using an instrument, single-nucleotide polymorphisms (SNPs), as a proxy for the exposure (9). In the MR study, we determined the SNPs as instrumental variables to assess the causality between oral health conditions and urolithiasis (Supplementary Table 5). We discovered a causal association forms urolithiasis to gingivitis and periodontal diseases [OR (95% CI), 1.174 (1.009–1.366), *p* = 0.038 in univariate MR and OR (95% CI), 1.207 (1.027–1.418), *p* = 0.022 in multivariable MR]. A causal link from urolithiasis to impacted teeth [OR (95% CI), 1.801 (1.086–2.988), *p* = 0.031] in bidirectional MR (Figure 3A). The effect of independent SNPs was shown in Figures 3B, C and Supplementary Figures 2A, B. We applied sensitivity analysis with the leave-one-out method (Supplementary Figures 2C, D).

## Discussion

This was the first study to investigate the association between urolithiasis and oral health conditions. We observed that presenting caries indicated a negative correlation with the risk for urolithiasis while presenting gingivitis and impacted tooth manifested to be positively associated with urolithiasis.

Furthermore, MR design is a new technique to explain causal inference and direction using SNPs as instrumental variables. Hence, we applied the MR method to the European population to confirm our discoveries from a cross-sectional study. We observed that genetically predicted gingivitis was associated with a higher risk of urolithiasis and causality from urolithiasis to impacted teeth.

Dental caries is a chronic and multifactorial disease. It is usually caused by the acid produced from the fermentation of oral microorganisms (13). However, the salivary calcium and phosphate ions can protect against such a process by remineralization on the tooth surface (14). Arvin et al. reported that the intake of calcium from drinking water could reduce the development of dental caries (15). In the meanwhile, Pratyusha et al. also demonstrated that deficiency of vitamin D and salivary calcium could increase the risk for dental caries (16). The most common type of kidney stone is calcium oxalate stone, followed by calcium phosphate, and calcium is the most common component in all stone types (17). Furthermore, calcium metabolism and vitamin D are indicated to be highly involved in urolithiasis. From the epidemiological perspective, intake and serum levels of vitamin D and calcium were evidenced to be positively associated with the risk of urolithiasis (18). From a pathophysiological point of view, high levels of vitamin D, especially the active metabolite calcitriol, could increase digestive calcium absorption and increase urine calcium excretion (19). Above all, deficiency of vitamin D and calcium might be a risk factor for caries and a protective factor for urolithiasis, which could explain the negative correlation between caries and urolithiasis. However, no causality was observed through MR analysis, and it might be attributed to insufficient statistical effect.

A periodontal disease which included gingivitis and periodontitis was reported to affect the supporting structures of the teeth and further contribute to systemic diseases. It was demonstrated that periodontal disease was linked to metabolic disorders, including diabetes mellitus, fatty liver, and obesity (20). Meanwhile, these factors were acknowledged to be the risk factor for the formation of kidney stones (21). Nevertheless, we adjusted for these factors, and oral inflammation was still positively correlated with the risk of urolithiasis, indicating that other crosstalk existed between periodontal disease and urolithiasis. The dissemination of microorganisms and virulence factors to distal sites through circulation and the interaction between the oral microbiome and inflammation activation were stated to induce various diseases (22). Other possible mechanisms of how periodontal disease affected other systems included cytokine communications, neuronal signals, and innate and adaptive immune responses (23). Therefore, oral inflammation could through similar pathophysiological processes contribute to urolithiasis. Estemalik et al. reported the detection of similar bacterial DNA in both the genitourinary system and oral cavity from the same individual (7). Our former publication also discovered the involvement of bacteria from the urinary tract in stone formation (24). These publications provided the possibility that oral microorganisms could migrate and colonize in the urogenital tract and contribute to stone formation. In addition, pre-inflammation cytokines, including IL-6, IL-17, IFN-γ, TNF-α, and other inflammatory molecules, were reported to elevate in the serum of periodontal disease patients (23). Kidney stone formation was highly linked to inflammation activation and immunology involvement according to Randall's plaque theory. Activation of the NLRP3 inflammasome, pro-inflammatory macrophages, and reactive oxygen species was highly involved in the process of stone formation (25). It can be speculated that the increased inflammatory factors in the peripheral blood caused by oral microbiota may affect the occurrence of stones. In addition, we unearthed interaction effects among the oral conditions on the stone formation. The aforementioned evidence gave an insight into the crosstalk between periodontal disease and urolithiasis. Nevertheless, explorations on concrete mechanisms of how periodontal disease interacted with urolithiasis were warranted.

Urolithiasis was detected to be associated with abnormal mineralization and osteogenic trans-differentiation of epithelial cells, indicating that a disorder of bone-related metabolism could elevate the risk of urolithiasis (26). Furthermore, during odontogenesis, dental follicle cells can differentiate into osteoblasts and can recruit and activate osteoclasts, which plays a significant role in tooth eruption (27). Hence, pathologic bone remodeling might induce an impacted tooth. In addition, enamel renal syndrome is an autosomal recessive disorder characterized by nephrocalcinosis and impacted posterior teeth with hyperplastic pericoronal follicles, which could provide evidence for the genetic association between urolithiasis and impacted teeth (28). Therefore, bone metabolism could build a bridge between urolithiasis and impacted tooth status.

According to our statistical analyses, we observed a synergistic effect of age and oral pathological conditions on stone formation risk. We discovered that aged individuals with poor oral health were more vulnerable to urolithiasis and the OR for urolithiasis of gingivitis increased by nearly 30% (from 1.648 to 2.106) as age increased from 29–44 to 44–59. An epidemiological study indicated that the incidence, prevalence, and severity of periodontitis were positively associated with age (29). A previous study also manifested that age could interact with oral health conditions through cellular senescence and the immune system (30). Moreover, in unerupted teeth, squamous metaplasia was observed to be associated with age, while inflammation in the dental follicle was described to be related to squamous epithelial metaplasia (31). On the other hand, according to epidemiological research on urolithiasis, age was demonstrated to be associated with the stone types and urinary biochemical parameters, and age-related risk comorbidities for urolithiasis (32). Given that aging showed a high correlation to both oral conditions and kidney formation, it would be more appropriate to pour more attention into the risk for urolithiasis in aged individuals with poor oral conditions.

This study still harbored several limitations. First, it was arduous to eliminate the bias since our analyses depended on single-center physical examination information. Second, the diagnosis of urolithiasis was based on the results of ultrasonography rather than computed tomography. Nevertheless, ultrasonography was recommended for screening in a large population (33). Third, no information on the stone composition was obtained. Accordingly, we adjusted UpH to eliminate the bias from stone types and urine parameters (34). Fourth, we lacked dietary information. Therefore, multicenter prospective studies and mechanism research were warranted in prospect. Fifth, no causality was observed between caries and urolithiasis, there could be unadjusted confounding, or the statistical effect was inadequate.

## Conclusion

This study was designed to elaborate on the association between oral health conditions and urolithiasis. The results cast new light on the risk factor and pathogenesis of kidney stone formation and could provide novel evidence for the oral–genitourinary axis and the systematic inflammatory network. Our findings could also offer suggestions for accurate clinical prevention strategies against stone diseases.

## Data availability statement

The original contributions presented in the study are included in the article/Supplementary material, further inquiries can be directed to the corresponding authors.

## Ethics statement

The studies involving human participants were reviewed and approved by the Institutional Review Board of Tongji Hospital, Tongji Medical College, Huazhong University of Science and Technology (Approval ID: TJ-C20160115). Written informed consent for participation was not required for this study in accordance with the national legislation and the institutional requirements.

## Author contributions

J-ZX, WG, and LC raised the conception and were responsible for the design of the study. J-XS, C-QL, Q-DX, J-LL, PZ, Y-ML, and YX took responsibility for the acquisition and integrity of the data and the data analysis. J-ZX, L-TM, S-HZ, W-JW, WG, and LC have interpreted the results. J-ZX and W-JW have drafted the manuscript. WG and YX provided intellectual content of critical importance to the study described, and all authors were involved in revising the manuscript critically. All authors read and approved the final manuscript and agreed to be accountable for all aspects of the study in ensuring that questions related to the accuracy or integrity of any part of the study are appropriately investigated and resolved.

## Supplementary material

Supplementary Figure 1 Illustrations of the interaction of BMI. Adjusted as Model 3 (see Methods–Statistical Analyses section for descriptions of Model 3). **(A)** Illustration showed the interaction between BMI and the gingivitis condition. **(B)** Illustration showed the interaction between BMI and the impacted tooth condition.

Supplementary Figure 2 **(A)** Scatter plots showed the effect of instrumental SNPs of gingivitis and periodontal diseases on urolithiasis. **(B)** Scatter plots showed the effect of instrumental SNPs of urolithiasis on impacted teeth. **(C)** Sensitivity analysis for the effect of gingivitis and periodontal diseases on urolithiasis applying the leave-one-out method. **(D)** Sensitivity analysis for the effect of urolithiasis on impacted teeth applying the leave-one-out method.

Supplementary Table 2 Association between oral health conditions and urolithiasis grouped by BMI.

Supplementary Table 3 Association between oral health conditions and urolithiasis grouped by sex.

Supplementary Table 4 Sensitivity analysis to characterize the association between oral health conditions and urolithiasis with missing values.
