# Supplementary figures and images for: Interconnections between urolithiasis and oral health: a cross-sectional and bidirectional Mendelian randomization study
Source: Front Med (Lausanne). 2023 Apr 26;10:1174502. doi: 10.3389/fmed.2023.1174502 (PMC10169673; doi:10.3389/fmed.2023.1174502)

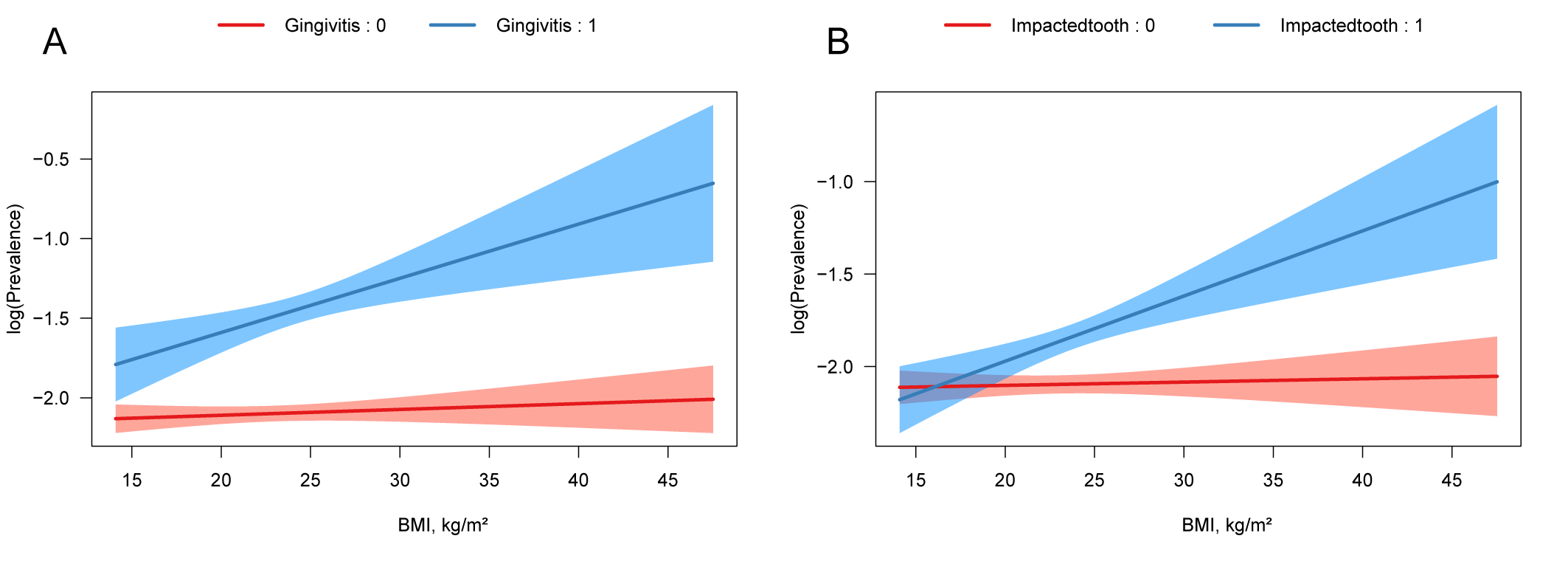

Supplement: Supplementary Figure 1 — Illustrations of the interaction of BMI. Adjusted as Model 3 (see Methods–Statistical Analyses section for descriptions of Model 3). (A) Illustration showed the interaction between BMI and the gingivitis condition. (B) Illustration showed the interaction between BMI and the impacted tooth condition. [file Image_1.TIFF]

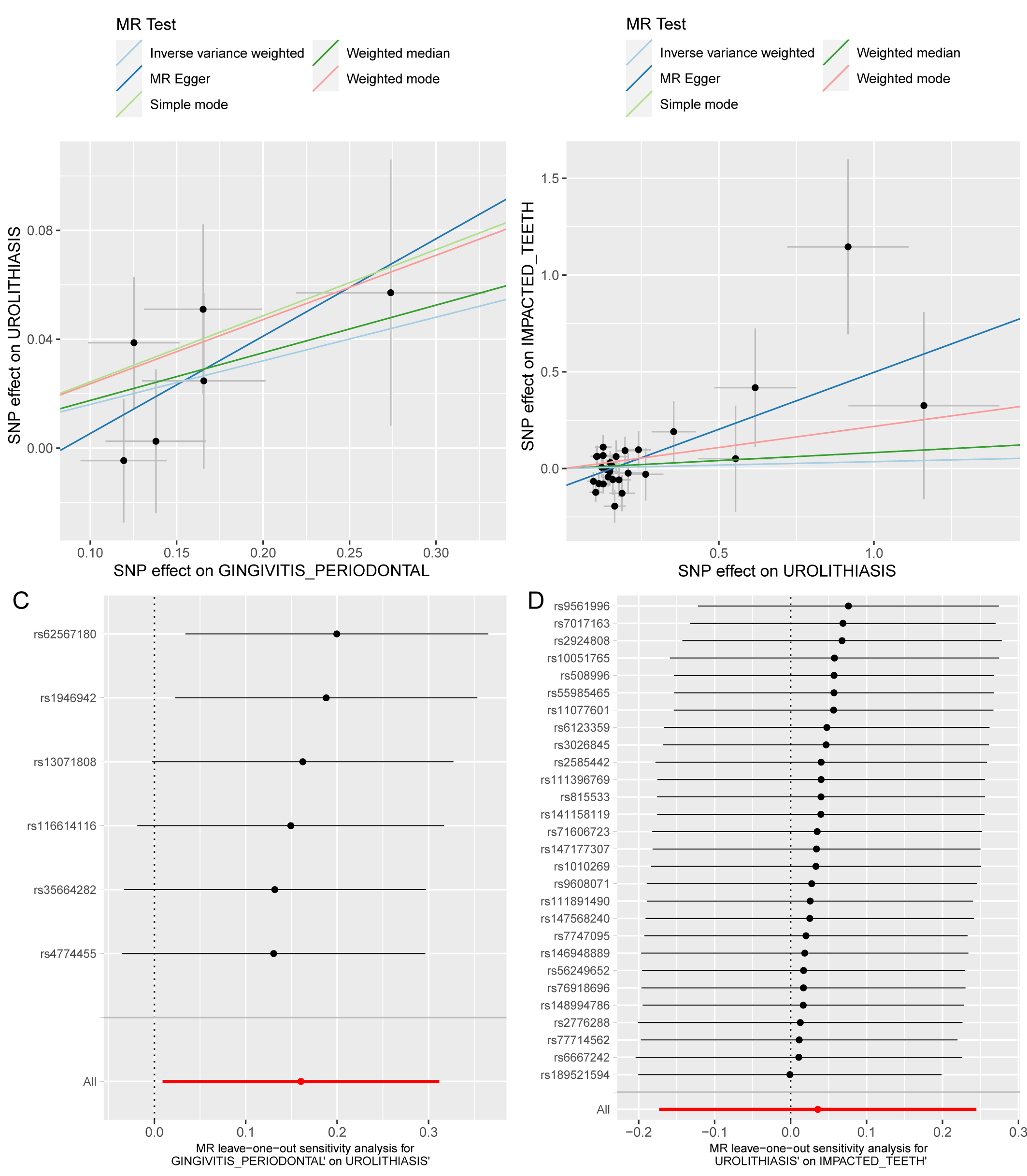

Supplement: Supplementary Figure 2 — (A) Scatter plots showed the effect of instrumental SNPs of gingivitis and periodontal diseases on urolithiasis. (B) Scatter plots showed the effect of instrumental SNPs of urolithiasis on impacted teeth. (C) Sensitivity analysis for the effect of gingivitis and periodontal diseases on urolithiasis applying the leave-one-out method. (D) Sensitivity analysis for the effect of urolithiasis on impacted teeth applying the leave-one-out method. [file Image_2.TIFF]
