# Supplementary material for: Interconnections between urolithiasis and oral health: a cross-sectional and bidirectional Mendelian randomization study
Source: Front Med (Lausanne). 2023 Apr 26;10:1174502. doi: 10.3389/fmed.2023.1174502 (PMC10169673; doi:10.3389/fmed.2023.1174502)
Supplement: Supplementary Table 2 — Association between oral health conditions and urolithiasis grouped by BMI. [file Table_1.DOCX]

**sTable 2. Association between oral health conditions and urolithiasis grouped by BMI.**

**Odds Ratio (95% CI)**

|  | BMI≤18.5 | 18.5<BMI≤23.9 | 23.9<BMI≤27.9 | 27.9<BMI | *p* for interaction |
| --- | --- | --- | --- | --- | --- |
| Caries | 0.838 (0.632 ~ 1.099) | 0.910 (0.843 ~ 0.982) * | 0.946 (0.869 ~ 1.030) | 0.835 (0.714 ~ 0.974) * | 0.170 |
| Residual Root or Crown | 1.016 (0.645 ~ 1.548) | 1.042 (0.933 ~ 1.162) | 1.047 (0.935 ~ 1.169) | 0.817 (0.666 ~ 0.995) * | 0.058 |
| Dental Calculus | 0.846 (0.643 ~ 1.105) | 1.024 (0.954 ~ 1.098) | 0.970 (0.900 ~ 1.046) | 0.980 (0.862 ~ 1.114) | 0.988 |
| Dental Plaque | 0.577 (0.260 ~ 1.141) | 0.984 (0.867 ~ 1.115) | 0.928 (0.834 ~ 1.032) | 0.903 (0.758 ~ 1.074) | 0.624 |
| Gingivitis | 1.390 (0.803 ~ 2.270) | 1.747 (1.554 ~ 1.960) *** | 2.038 (1.819 ~ 2.281) *** | 2.265 (1.865 ~ 2.744) *** | 0.005 ** |
| Impacted Tooth | 0.790 (0.545 ~ 1.119) | 1.283 (1.164 ~ 1.413) *** | 1.392 (1.251 ~ 1.548) *** | 1.345 (1.111 ~ 1.623) ** | <0.001 *** |

Abbreviations: BMI, body mass index; CI, confidence interval.

Model 3 was applied (see Methods-Statistical Analyses section for descriptions of models 3).

* *p*<0.05; ** *p*<0.01; *** *p*<0.001.

**sTable 3. Association between oral health conditions and urolithiasis grouped by sex.**

**Odds Ratio (95% CI)**

|  | Female | Male | *p* for interaction |
| --- | --- | --- | --- |
| Caries | 0.904 (0.828 ~ 0.986) * | 0.916 (0.857 ~ 0.978) ** | 0.929 |
| Residual Root or Crown | 0.996 (0.872 ~ 1.135) | 1.014 (0.930 ~ 1.104) | 0.979 |
| Dental Calculus | 0.985 (0.904 ~ 1.073) | 0.993 (0.939 ~ 1.050) | 0.672 |
| Dental Plaque | 1.530 (0.240 ~ 5.512) | 0.939 (0.871 ~ 1.012) | 0.559 |
| Gingivitis | 2.028 (1.733 ~ 2.362) *** | 1.909 (1.755 ~ 2.075) *** | 0.471 |
| Impacted Tooth | 1.159 (1.013 ~ 1.322) * | 1.361 (1.261 ~ 1.468) *** | 0.037 * |

Abbreviations: CI, confidence interval.

Model 3 was applied (see Methods-Statistical Analyses section for descriptions of models 3).

* *p*<0.05; ** *p*<0.01; *** *p*<0.001.

**sTable 4. Sensitivity analysis to characterize the association between oral health conditions and urolithiasis with missing values.**

**Odds Ratio (95% CI)**

|  | Model 1-adjusted | Model 2-adjusted | Model 3-adjusted |
| --- | --- | --- | --- |
| Caries | 0.907 (0.861 ~ 0.955) *** | 0.919 (0.872 ~ 0.969) ** | 0.908 (0.858 ~ 0.960) *** |
| Residual Root or Crown | 1.211 (1.129 ~ 1.297) *** | 0.989 (0.919 ~ 1.063) | 0.989 (0.916 ~ 1.066) |
| Dental Calculus | 1.134 (1.083 ~ 1.187) *** | 0.983 (0.938 ~ 1.031) | 0.984 (0.937 ~ 1.035) |
| Dental Plaque | 1.492 (1.393 ~ 1.597) *** | 0.929 (0.863 ~ 0.999) * | 0.945 (0.876 ~ 1.019) |
| Gingivitis | 2.247 (2.090 ~ 2.414) *** | 1.937 (1.799 ~ 2.085) *** | 2.021 (1.866 ~ 2.187) *** |
| Impacted Tooth | 1.305 (1.224 ~ 1.390) *** | 1.311 (1.226 ~ 1.400) *** | 1.312 (1.219 ~ 1.411) *** |

Multiple imputation method was applied to process the missing values. Details could be found in methods section.

Models:

Model 1: adjusted for oral health conditions (caries, residual root or crown, dental calculus, dental plaque, gingivitis, and impacted tooth).

Model 2: model 1 plus demographic characteristics and comorbidities (age, sex, BMI, HBP, DM, CHD, and FL).

Model 3: model 2 plus laboratory outcomes (Alb, Glo, eGFR, HDL, LDL, TG, UA, Glu, and UpH).

Abbreviations: BMI, body mass index; HBP, hypertension; DM, diabetes mellitus; CHD, Coronary heart disease; FL, fatty liver; Alb, albumin; Glo, globulin; SCr, serum creatinine; eGFR, estimated glomerular filtration rate; HDL, high-density lipoprotein cholesterol; LDL, low-density lipoprotein cholesterol; TG, triglycerides; UA, uric acid; Glu, fasting glucose; UpH, Urine pH; CI, confidence interval.

* *p*<0.05; ** *p*<0.01; *** *p*<0.001.
